# Integrative Assessment of Seminal Plasma Biomarkers: A Narrative Review Bridging the Gap between Infertility Research and Clinical Practice

**DOI:** 10.3390/jcm13113147

**Published:** 2024-05-27

**Authors:** Efthalia Moustakli, Athanasios Zikopoulos, Charikleia Skentou, Sofoklis Stavros, Nikolaos Sofikitis, Ioannis Georgiou, Athanasios Zachariou

**Affiliations:** 1 Laboratory of Medical Genetics, Faculty of Medicine, School of Health Sciences, University of Ioannina, 45110 Ioannina, Greece; igeorgio@uoi.gr; 2 Obstetrics and Gynecology, Royal Devon and Exeter Hospital, Barrack Rd, Exeter EX 25 DW, UK; thanzik92@gmail.com; 3 Department of Obstetrics and Gynecology, Medical School of Ioannina, University General Hospital, 45110 Ioannina, Greece; haraskentou@gmail.com; 4 Third Department of Obstetrics and Gynecology, Attikon Hospital, Medical School, National and Kapodistrian University of Athens, 12462 Athens, Greece; sfstavrou@med.uoa.gr; 5 Department of Urology, School of Medicine, Ioannina University, 45110 Ioannina, Greece; nsofikit@uoi.gr (N.S.); zahariou@otenet.gr (A.Z.)

**Keywords:** semen quality, seminal biomarkers, male fertility, infertility treatment, clinical diagnosis, assisted reproductive technology (ART), reproductive health

## Abstract

Infertility represents a significant global health challenge impacting millions of couples worldwide. Approximately half of all infertile couples exhibit compromised semen quality, indicative of diminished male fertility. While the diagnosis of male infertility traditionally relies on semen analysis, its limitations in providing a comprehensive assessment of male reproductive health have spurred efforts to identify novel biomarkers. Seminal plasma, a complex fluid containing proteins, lipids, and metabolites, has emerged as a rich source of such indicators. Reproduction depends heavily on seminal plasma, the primary transporter of chemicals from male reproductive glands. It provides a non-invasive sample for urogenital diagnostics and has demonstrated potential in the identification of biomarkers linked to illnesses of the male reproductive system. The abundance of seminal proteins has enabled a deeper understanding of their biological functions, origins, and differential expression in various conditions associated with male infertility, including azoospermia, asthenozoospermia, oligozoospermia, teratozoospermia, among others. The true prevalence of male infertility is understated due to the limitations of the current diagnostic techniques. This review critically evaluates the current landscape of seminal plasma biomarkers and their utility in assessing male infertility. Βy bridging the gap between research and clinical practice, the integrative assessment of seminal plasma biomarkers offers a multimodal approach to comprehensively evaluate male infertility.

## 1. Introduction

Investigating the physiology of humans and seminal plasma (SP) biomarkers has emerged as a crucial area of research, providing insight into the intricate relationships among male reproductive health, fertility outcomes, and overall wellbeing [1]. Seminal plasma, a complex fluid containing a diverse array of proteins, lipids, hormones, and other bioactive compounds, is a vital medium that supports and controls several elements of the male reproductive process. Βiomarkers in this milieu can help diagnose, predict, and treat reproductive health problems [2]. They serve as molecular indicators of physiological states, pathological disorders, or responses to environmental factors [3].

Comprehending groundbreaking plasma biomarkers goes beyond simply explaining male reproductive function. It reveals perspectives into more intricate health paradigms, including immunological regulation, oxidative stress dynamics, and endocrine background [4]. In addition, SP facilitates the transmission of vital information from the male reproductive system to the female reproductive system, influencing fertilization, pregnancy establishment, and embryo development [2]. Therefore, SP biomarkers are essential for offspring health and reproduction success in men, serving as indicators of the state of the male reproductive system [5].

Epidemiological research suggests that rising rates of male infertility in some areas may be caused by sedentary lifestyles, environmental pollutants, and changes in reproductive health patterns. It is critical to understand the risk factors associated with male infertility in order to make informed decisions about diagnosis, treatment, and prevention. Complex interactions among several risk variables may have an impact on reproductive health. Male reproductive health can be adversely affected by lifestyle choices like drug misuse, obesity, and inadequate nutrition as well as environmental pollutants. 

This narrative review delves deeply into the various functions, diagnostic potential, and clinical implications of innovative plasma biomarkers in human physiology. The study aims to illuminate the usefulness of SP biomarkers as markers of male fertility, reproductive diseases, and general health issues by piecing together results from seminal studies and innovative research initiatives. For a thorough understanding of the molecular landscape of seminal plasma, proteomics, metabolomics, and genomes can be combined. Potential biomarkers with greater predictive ability for the diagnosis and treatment of infertility could be found through future study using machine learning algorithms and bioinformatics technologies.

## 2. Physiologic Aspects of Seminal Plasma

**Figure 1:**
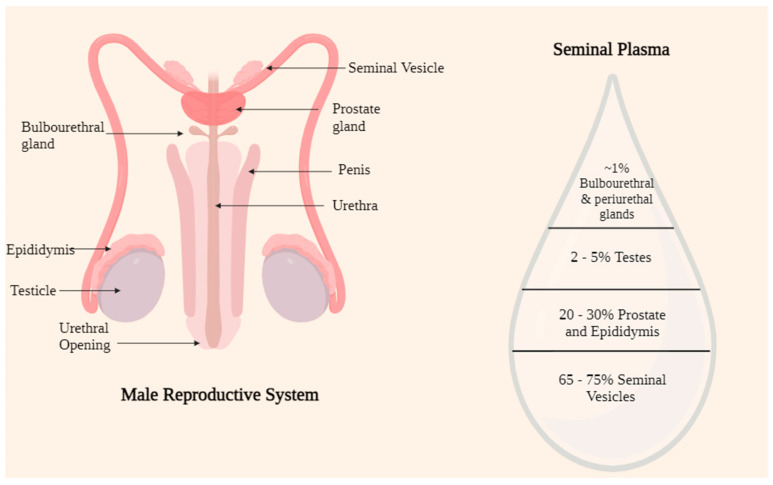
An illustration of the seminal plasma’s composition and the male reproductive system.

To produce semen, glands in the male urogenital tract secrete a fluid known as SP. It comprises lipids, glycans, inorganic ions, small molecule metabolites, and biopolymers such as cell-free DNA, RNA, microRNAs, peptides, proteins, and oligosaccharides [1]. Zinc and selenium are essential building blocks of antioxidant enzymes crucial for proper spermatogenesis. The seminal vesicles contribute the majority of the molecules found in SP [1]. Additionally, the prostate releases a fluid that contains lipids, citrate, and proteolytic enzymes to control sperm maturation, semen liquefaction, and motility [2]. The bulbourethral glands release mucin, sialic acid, and galactose to lubricate the semen and improve the effectiveness of sperm transmission. Putrescine, spermine, and spermidine are polyamines that keep semen’s pH alkaline [3]. However, further research is required to ascertain their diagnostic efficacy (Figure 1).

Seminal plasma not only carries and nourishes sperm cells during conception, but also influences their interaction with the female reproductive system [4]. Previous research suggests a limited impact due to its inability to penetrate cervical mucus. However, new research indicates that its constituents play a significant and active role in fertilization [5]. Serum plasma contains immunomodulatory substances that facilitate successful fertilization and early embryonic development by preventing the female immune system from attacking sperm [6]. Studies by Baker and Bellis, among others, offer compelling evidence that SP actively participates in fertilization rather than merely serving as a passive medium [7]. Consequently, health professionals should consider the impact of SP components on sperm motility, transport, and interactions within the reproductive tract (Table 1).

## 3. Seminal Plasma’s Immunological Apparatus 

Allogenic spermatozoa and embryos may be recognized and accepted by the female immune system if SP immune system components are present. Studies have identified 24 putative biomarkers associated with the immune system, including cytokines, immune cells, and antigens [8]. Men with leukocytospermia, characterized by too many white blood cells in the sperm, often produce sperm with low motility, making fertilization challenging, even with assisted reproductive technology (ART). Membrane-bound CD glycoproteins were used to detect immune cells, while immunohistochemistry was employed to find leukocytes and subpopulations in SP. High-fertilization groups exhibited considerably increased levels of SP CD14, a marker of monocytes and macrophages [9]. Various immune-competent cells in the male reproductive tract produce pro- and anti-inflammatory cytokines. In women exposed to SP during in vitro fertilization (IVF) and/or intracytoplasmic sperm injection (ICSI) treatment, three studies have found no discernible change in seminal TGF-β1 concentration between successful and unsuccessful pregnancies. It should be noted, however, that there may be an overlap in demographic data and research on population recruitment.

Research has focused on exploring the connection between IVF fertilization rates and SP cytokines. The group that achieved pregnancy showed a greater transforming growth factor beta 1 (TGF-β1) to interleukin 18 (IL-18) ratio (TGF-β1/IL-18), despite having much lower pro-inflammatory IL-18 levels compared to those who did not conceive [10] According to research by Seshadri et al. (2011), there were no significant differences in SP sHLA-G concentrations between men whose partners became pregnant and those whose partners did not. Nevertheless, men with a high fertilization rate (≥60%) appeared to have significantly higher levels of SP IL-11 compared to males with a low rate of fertilization (<35%) [11]. Studies have linked reduced fertility and poor ART outcomes with the homozygous HLA-G genotype. Nonetheless, other trials did not reveal significant correlations between SP and the time taken to conceive following IVF [12,13].

Sperm surface antigens can be recognized by immune cells as alien, and in reaction, they can form anti-sperm antibodies (ASA). ASA has been detected in SP and may have an effect on sperm motility, quality, and capacitation [14]. In a study by El-Sherbiny et al. (2021), which compared men who tested positive or negative for ASA in their SP, the variations in fertilization rates and clinical pregnancy success following ICSI were assessed [15]. Infertile men more frequently present high levels of s-EI in SP, indicating genital tract inflammation. However, fertilization and pregnancy rates do not significantly correlate with SP ASA levels [16].

## 4. Metals, Trace Elements and Nucleic Acids

Male fertility is significantly impacted by heavy metals, especially trace metals. Technology for assisted reproduction may benefit from the usage of stem cells. Socioeconomic factors affecting population demography are influenced by heavy metal contamination. Although they are not necessary, heavy metals are extremely harmful to humans. Acute toxicity is not common. Human development and reproduction may be adversely affected by prolonged exposure to Cd, Pb, As, and Hg. In clinical practice, heavy metal levels are measured in the blood and urine. Urine tests react to the concentration of heavy metals in urine, whereas blood tests identify their retention in the circulatory system [17].

Pollution has led to an increase in exposure to dangerous heavy metals from the environment, which may interfere with spermatogenesis, endocrine function, sperm quality, and fertilization ability [18]. Zinc, iron, and magnesium are trace elements necessary for male reproduction. Five research studies have identified 25 putative biomarkers for ART outcomes. While two studies found no significant link between lead (Pb) and IVF fertilization rate, four studies found a negative correlation [10]. A thorough analysis of many metals and trace elements in SP was conducted by Rodríguez-Díaz et al. in 2022. They found no connections with live births, clinical pregnancy, or fertilization rates. The fertilization rate, being a continuous variable; showed a significant negative correlation with vanadium (V) concentration in the group with high levels [19]. The analysis revealed adverse effects on blastocyst development and quality. Conversely, no meaningful relationship was observed between fertility, pregnancy, or the number of live births and arsenic (As), barium (Βa), chromium (Cr), copper (Cu), mercury (Hg), manganese (Mn), molybdenum (Mo), nickel (Ni), and thallium (Ti) [19].

The body absorbs cadmium through food, drink, and the atmosphere; it is not a necessary element for human health. Regarding male infertility, it is truly detrimental [20]. According to research by Chabchoub et al., aberrant sperm shape and urine cadmium levels were positively connected. It was discovered that infertile women had higher levels of Pb and As than pregnant ones. All variables were taken into account and blood and urine Cd levels were not associated with infertility [21]. 

Three trials identified seven sperm nucleic acids as possible indicators for ART outcomes. Among the several mRNAs found in SP are Fertilin-β (ADAM-2), and protamines 1 and 2 (PRM1, PRM2) mRNA expressions [13]. In a study by Sukhikh et al. (2012), participants were categorized into three groups: IVF failure, spontaneous miscarriages before week 11, and biochemical pregnancy [22]. Couples experiencing IVF and embryo transfer program failure had lower levels of ADAM-2 mRNA expression in SP. There were significant decreases in the subgroups experiencing biochemical gestation and IVF failure, as well as miscarriage earlier than week 11 [22]. Protamines are essential for the modification of chromatin and ADAM-2 is necessary for gamete fusion, which affects chromatin condensation and embryo survival. During the division of zygote and embryo, proteins facilitate interactions between the chromosomes and the male and female genomes. Compared to the control group, PRM1 and PRM2 expression dramatically decreased in the biochemical gestation and control groups, but not in the spontaneous abortion group (miscarriage before week 11) [22].

## 5. Male Infertility and Seminal Plasma Indicators

Researchers are investigating markers found in SP to better understand male infertility and investigate potential therapy approaches [1]. The SP proteome provides valuable insights into the function of proteins in male reproduction. As sperm cells mature, a protein known as TEX101 is broken down, producing fertile spermatozoa [23]. Additionally, the epididymis secretes the protein ECM1, a reliable marker for distinguishing between obstructive and non-obstructive azoospermia, with data indicating 81% sensitivity and 100% specificity [24]. By incorporating these biomarkers into diagnostic processes, clinicians can offer more precise treatment strategies for infertile men.

The lipocalin-type prostaglandin D synthase (L-PGDS) protein envelops spermatozoa in the male genital tract [25]. This is believed to facilitate the transportation of thyroid hormones, retinoids, and vital fatty acids to promote the growth and maturity of germ cells, although its precise function remains uncertain. In males with azoospermia, the presence of L-PGDS in SP may serve as a biomarker for assessing the patency of the seminal tract [26]. Additionally, studies have revealed that the absence of lactate dehydrogenase (LDHC) isoform C severely affects sperm motility, highlighting the vital function that LDHC-mediated glycolysis plays in providing energy for this process [27].

Men can also be classified as fertile or infertile using markers like glucose-like protein 1 (TKTL1) and phosphoglycerate kinase 2 (PGK2) [28,29]. The mammalian epididymis has a ubiquitin-dependent system that regulates sperm quality and male fertility. Ubiquitin is another strong biomarker that can assess male fertility and human semen quality without requiring a biopsy [30]. Furthermore, elevated levels of sperm-specific protein (SPSP) have been associated with decreased sperm shape and count.

Infertility may result from the presence of oxidized free radicals in SP. Male infertility frequently results in lower amounts of free sulfhydryl and overexpression of membrane metalloendopeptidase [31]. The activity of SP superoxide dismutase (SOD), however, has been positively linked to sperm motility and concentration [32]. Low fertility rates are also associated with the secretory β-galactoside binding protein known as galactin-3 [33]. According to one study, infertile males treated with antioxidant supplements high in SOD, demonstrated significant reductions in sperm DNA fragmentation, according to a study [34]. The epithelial cells of the prostate gland release PSA, a serine protease essential for sperm motility and liquefaction [35]. PSA levels vary between 0.5 and 2 ng/mL. Further research is required to fully grasp its role in male fertility [36].

One of the precursor forms of the sperm motility inhibitor seen in human seminal plasma comes from seminal vesicles. Prostatic proteases break down this precursor into smaller peptides soon after ejaculation. Combining C4 reverse-phase high-performance liquid chromatography with S-Sepharose cation-exchange chromatography allowed for the purification of the seminal plasma sperm motility inhibitor (SPMI) precursor. The protein known as semenogelin, which is considered to be the primary structural element of semen coagulum, was found to have an amino acid makeup that was nearly exact. Protease prostate-specific antigen (PSA) has the ability to break it down in a way that is similar to how complete semen breaks down. The findings indicate that intact semenogelin, which makes up the majority of seminal vesicles and the coagulum, has the ability to immobilize spermatozoa.

The biological function of protease prostate-specific antigen (PSA) is to dissolve cervical mucus, release motile spermatozoa from semen, and facilitate sperm entry into the uterus. It is uncertain how PSA is distributed in both fertile and infertile males. The PSA distribution did not differ between the two groups, according to Mifsud et al. The concentration of total PSA and the percentage of PSA > 1 ng/mL were higher in infertile males. Serum PSA levels were independently predicted by infertility status.

Advanced plasma biomarkers have the potential to outperform or at least supplement existing imaging modalities and blood-based testing for fertility [37]. While ultrasonic and magnetic resonance imaging are the most promising noninvasive imaging methods, there are barriers to their widespread use [38]. Analyzing biomarkers in SP presents a more desirable method. One benefit of this is approach is that it avoids the need for direct access to systemic circulation. Moreover, modern imaging technologies are still inaccessible in isolated locations and are not easily accessible in smaller healthcare facilities [39].

Seminal plasma biomarkers have the potential to enable long-term tracking of disease development or recurrence. They may also be more cost-effective than some current diagnostic techniques [10]. They are easily collected and transferred for processing, even in resource-constrained contexts, and can yield helpful diagnostic data. However, further research and development are required before the full diagnostic potential of these biomarkers can be realized [40]. 

## 6. SARS-CoV2, Oxidative Stress, and Male Infertility

The reproductive system is just one aspect of peoples’ lives and health that has been significantly impacted by SARS-CoV-2. The virus’s mechanism of cellular entry has been identified as COVID-19, and it has been identified that it affects male fertility [41]. The virus may interfere with the regulation of gonadotropin secretion, which would cause a decrease in testosterone levels. The hypothalamic–pituitary–gonadal axis links the brain and testes [42]. Although the testicles have a unique immunity due to the blood–testes barrier, oxidative stress and inflammation can damage their biological elements. Oxidative stress has been connected to COVID-19 and has the potential to hinder spermatogenesis and testosterone synthesis [43].

Entering host cells, the SARS-CoV-2 virus uses the membrane-bound angiotensin-converting enzyme 2 (ACE2), a crucial component of the renin–angiotensin system (RAS), as a human gate [44]. The peptide Ang1-7, which has anti-inflammatory, anti-fibrotic, and vasodilatory qualities, is generated when ACE2 breaks angiotensin-2 (AngII) [45]. Abnormal expression of genes related to mitochondrial function and testicular steroidogenesis has been observed in animal models with RAS component loss, along with enhanced expression of ACE2 in the testicles and epididymis. It is not unexpected that COVID-19 affects male fertility because endothelial cells produce ACE2, and male fertility is known to be impacted by endothelial dysfunction and inflammation. Normal physiological functions, such as hormone balance and temperature regulation, can be affected by inflammation. Individuals who suffer from systemic inflammation exhibit increased levels of luteinizing hormone (LH) and low ratios of testosterone to LH. Hyperintense signals on neuroimaging can be a symptom of hypothalamic issues as well as an enlarged pituitary gland [41].

Oxidative stress (OS) arises from an imbalance between the antioxidant capacity of the sperm and the generation of reactive oxygen species (ROS) and is a common cause of male infertility [46]. Single- and double-strand DNA breaks, as well as oxidative nucleotides, can produce oxidative DNA damage (OS), which can lead to male infertility [47]. Male infertility has a significant diagnostic potential [48]. To measure oxidative DNA damage, 8-hydroxy-2’-deoxyguanosine (8-OHdG) can be used as a biomarker. The presence of OS in spermatozoa can result in damage to the cell membrane, which can hinder the sperm–oocyte union by decreasing membrane fluidity [49]. Malondialdehyde (MDA) is a detectable result of lipid peroxidation that may be used as an OS marker. Studies have shown a negative relationship between seminal MDA levels and the percentage of fertilized oocytes [50]. Although Ahelik et al. (2015) found a strong correlation between the pregnancy rate and OS in male partners, there were insufficient data to draw firm conclusions [51].

The entire ejaculate’s ROS levels had a diagnostic value that varied from poor to exceptional. Three methods for determining total antioxidant capacity (TAC) and oxidative reduction potential (ORP) were also examined [52]. Forty percent of male infertile men have been shown to have elevated levels of ROS in their SP [53]. In SP, twelve potential OS biomarkers were covered in eight publications. Two studies evaluated the relationship between ROS levels in SP and the results of IVF/ICSI [54,55,56]. There is no significant correlation observed between the rate of IVF fertilization and SOD, glutathione reductase, glutathione peroxidase (GPX), or SP catalase-like activity. Sperm lose their cytoplasmic enzymes during development and transit [57]. Combining the IVF and ICSI groups revealed a substantial negative association. However, research has not discovered any noteworthy correlations between levels of hydrogen peroxide (H_2_O_2_) in SP and outcomes of IVF, biochemical pregnancy, clinical pregnancy, or live birth rates [54].

## 7. Indicators of the Effectiveness of Assisted Reproductive Technologies (ART)

A successful ART procedure depends on the quality of the sperm, since healthy sperm can improve pregnancy outcomes and early embryonic development. In contrast, higher mRNA expression in sperm enhances the chance of retrieving viable embryos. Decreased expression of miR-149 in sperm can suggest a high incidence of high-quality embryos following IVF treatment. Since they are strongly correlated with the kinetics of embryonic development and clinical results, miR-34c-5p and hsa-mir-191 are interesting biomarkers for evaluating sperm DNA fragmentation (SDF) and ART outcomes [58]. 

The number of embryos developed may be positively impacted by the overexpression of these miRNAs, which may govern cleavage-stage embryo development. In spent culture medium (SCM) of G1-, G2-, and G3-grade embryos with sperm, the expression levels of miR-19b-3p and miR-let-7a-5P can be compared to potentially identify biomarkers for predicting pregnancy outcomes [58,59]. When cryopreserved sperm is used to fertilize IVF embryos and frozen–thawed sperm, higher expression levels of these biomarkers may increase the success rate of ART and decrease the rates of embryo development. Overall, the expression of specific miRNAs in sperm (miR-149, hsa-mir-191, miR-19b-3p, miR-let-7a-5p, miR-34c, miR-140, miR-21, and miR-375) may be a useful predictor of the quality of the embryo and the success of pregnancy [60].

Despite the use of intrauterine insemination (IUI), in vitro fertilization (IVF), and ICSI to improve conception in couples, the success rate of ART is still low. The IVF outcomes and the DFI have little to no link, and the ASA test does not considerably increase the accuracy of predicting spontaneous conception rates [57]. The oxidative environment that ROS in semen might give sperm may aid in predicting how well ART treatments would work. Even so, a study discovered comparable rates of fertilization, even though the IVF groups had observably higher ROS concentrations. The role of reactive oxygen species (ROS) and how antioxidant therapy affects the results of assisted reproduction need more research. Approximately 22.22% of ART regimens are successful overall [5].

The selection of healthy sperm is essential for successful ART fertilization. However, additional elements may be a factor in ineffective fertilization. These characteristics can include complex immunological and metabolic processes in addition to spermatozoa morphology [61]. More investigation is necessary to completely comprehend the intricate relationships among ROS, SP proteins, and sperm morphology and how these relationships affect assisted reproductive outcomes.

## 8. Biomarkers for the Grade and Outcome of Varicocele Surgery

The development of sperm is impacted by the increase in testicular temperature brought on by a varicocele. For every degree the temperature of the testicles rises, the number of sperm is thought to decrease by 40%. Male infertility is most commonly caused by a low sperm count. Sperm DNA may be affected by a varicocele.

Differentiating between individuals who are fertile and those who are infertile can be difficult, even though varicocele is a prevalent cause of sterility. Male fertility is adversely affected by varicocele through a number of processes. Sperm concentration, total sperm motility, and good sperm morphology are all positively correlated with low expression of seminal miR-122/-181a/-34c5, which is also linked to greater varicocele grades and bilateral varicocele [62]. With a negative correlation to both sperm count and seminal inhibin-B, seminal plasma miR-210-3p is a viable biomarker for predicting aberrant semen quality brought on by varicocele [57]. Additionally, varicocele is associated with elevated levels of other miRNAs that regulate the innate immune system and apoptotic signaling pathways. These miRNAs are interesting biomarkers for varicocele identification in clinical situations [63]. 

The two main causes of sperm destruction associated with varicocele are hyperthermia and oxidative stress. Patients with varicocele exhibit considerably reduced expression of miR-15a in their sperm, which is inversely correlated with heat shock protein A1B (HSPA1B) [64]. The levels of MiR-21/-34a/-122a are reduced in varicocele spermatozoa of varicocele that contain abnormal sperm. Semen samples from varicocele males had higher levels of oxidative stress than samples from fertile men. Moreover, varicocele patients have greater expression of miR-192a in their testicular and SP tissues following surgery, indicating that this biomarker may be useful in clinical settings for predicting varicocelectomy benefits [65]. In general, the degree of the disease, the number of sperm, and the results of surgery in patients with varicocele are correlated with the expression of different genes in seminal fluid.

## 9. Innovative Biomarkers for Sperm Retrieval and Non-Obstructive Azoospermia

Testicular spermatogenesis is compromised in non-obstructive azoospermia (NOA), resulting in the absence of sperm in semen. Testicular biopsies are useful in the diagnosis of NOA, but they have major drawbacks, including complications and the lack of active foci. Not all NOA patients are able to obtain sperm utilizing micro-TESE. ICSI is a method of choice for successful fertilization in such cases, but positive results are not guaranteed [62].

A predictive strategy for selecting appropriate individuals for micro-TESE was developed in one study using four mRNAs. Three miRNAs were discovered in SP and were shown to be downregulated in patients who had unsuccessful sperm retrieval (USR) as opposed to those who had successful sperm retrieval (SSR). These findings may provide non-invasive indicators for predicting testicular sperm retrieval outcomes [65]. Prior to undertaking a targeted biopsy, patients with azoospermia may benefit from using markers that indicate the existence of spermatozoa, such as two miRNA families that control spermatogenesis in mice. When compared to normal men, the expression of miR-34b/c and miR-449 is markedly downregulated in sickle cell only syndrome (SCOS), mixed atrophy, and germ cell arrest. According to these results, biomarkers may be used to forecast the course of male infertility [66].

Presently, there are notable disparities within the existing literature concerning the significance of clinical and biochemical predictors in determining sperm retrieval outcomes in NOA men. To address this gap, multicenter trials are imperative. For the non-invasive diagnostic of NOA, miRNAs in semen and SP can be useful. For instance, ITPR1 and miR-34b-5p might be useful biomarkers to anticipate azoospermia [67]. Diagnostic accuracy for identifying various NOA types can be increased by combining these miRNAs with other established testing. According to the study, there is a possibility to differentiate between fertile controls and NOA patients using different biomarkers in SP. Patients with NOA were found to have higher mean blood plasma levels, which were found to be adversely linked with both left and right testicular size and serum total testosterone. Alternatively explained, the most plausible diagnostic markers for NOA include miR-10b-3p, miR-34b-5p, miR-122, miR-31-5p, miR-141, miR-429, miR-7-1-3p, hsa-miR-34b*, miR-19b, miR-let-7a, miR−192a, miR-34c-5p, miR-146b-5p, miR-181a, miR-374b, miR-509-5p, miR-513a-5p, and miR-20a-5p [62]. 

## 10. Current Status of Male Infertility Assessment and Indexes for Further Male Infertility Biomarkers

The sex-chromosome aneuploidy known as Klinefelter syndrome (KS) varies in phenotypic features and symptom intensity. The emergence of KS might be influenced by epigenetic factors. Patients with KS exhibit substantial downregulation of miR-3648 and miR-3687, suggesting their role in immunological and metabolic problems [68]. The use of next-generation sequencing allowed Finocchi and Ibarra-Ramírez (2020) to identify aberrant expression of 166 miRNAs in target tissue samples from 200 subjects (40 KS, 60 non-obstructive azoospermia with normal karyotype, 60 obstructive azoospermia, 40 normozoospermic), 4 KS patients, and 5 control individuals with obstructive azoospermia, respectively [69,70]. The peripheral blood mononuclear cells (PBMCs) of patients with and without disease showed higher expression of 71 and lower expression of 18, respectively, according to differential expression analysis of mRNA expression [71].

Research on the significance of miR-3687 in infertility is still in its infancy, despite the fact that information on miR-3687’s involvement in reproductive processes is ongoing. MiR-3687 belongs to a class of short non-coding RNA molecules called microRNAs (miRNAs) that regulate the post-transcriptional control of gene expression. They play a key role in a number of biological processes that are essential for normal reproductive function, including differentiation, apoptosis, and cell proliferation. 

Though there may not be as many specific studies on miR-3687 and infertility, research exploring the broader role of miRNAs in reproductive health offers valuable insights. Dysregulation of miRNAs has been connected to a number of reproductive diseases, including endometriosis, polycystic ovarian syndrome (PCOS), male and female infertility, and recurrent pregnancy loss. 

The first stage of the fertility assessment method involves analyzing the sperm count, morphology, and motility of the semen. Additional investigations such as sperm chromatin evaluation, acrosome response, ROS, and chemical testing of SP are also feasible [72]. It is essential to perform several analyses in order to have a more complete understanding of fecundity. For consistent and dependable outcomes, standardized methods for data collection must be followed.

The possibility of pregnancy can be inferred from semen analysis, although male fertility is not correlated with it. Several sperm quality assays, such as the anti-sperm antibody (ASA) test, have been developed for thorough investigation. However, the therapeutic effects of ASA remain a subject for debate [29]. Male infertility and the DNA fragmentation index (DFI), a commonly used test for evaluating sperm DNA damage, do not consistently show correlation in some research. One major obstacle is the absence of consensus on the DFI reference range [73]. The World Health Organization (WHO) recommends establishing internal reference ranges based on specific characteristics of each laboratory or healthcare facility. According to the American Society of Reproductive Medicine (ASRM), routine DFI testing is not currently recommended for infertile couples [74].

Spermatozoa can be harmed by high ROS levels in seminal fluid, particularly in male infertile individuals. However, it is not recommended to remove all ROS because minimal levels are needed for crucial fertilization stages [75]. Genetic imbalances that contribute to issues with conception or implantation have been identified through fluorescence in situ hybridization (FISH) testing for sperm aneuploidy. Clinicians employ FISH for particular clinical situations, including couples experiencing recurrent miscarriages. To assess the sperm–mucus interaction, additional in vitro or post-coitally testing may be helpful [76].

## 11. Transcriptomics, Proteomics, Metabolomics

Compared to sperm, seminal plasma has a higher concentration of miRNA markers of semen quality. Noncoding RNAs, such as miRNAs and piRNAs, are essential regulatory elements in the processes of reproductive biology. Noncoding RNA (miRNAs and piRNAs) in semen provides enormous diagnostic value for problems with sperm quality [77]. These biomarkers offer potential to diagnose male reproductive issues more rapidly and accurately than current methods. Assessment simplicity, safety, effectiveness rate, usability variability, and consistency are the criteria used to evaluate diagnostic markers. According to studies, miRNAs may function as biomarkers for the diagnosis, prognosis, and response to therapy of diseases [78].

These RNAs have been shown through research to potentially function as biomarkers for problems associated with reproduction or the effectiveness of necessary procedures. All RNAs can be validated using quantitative reverse transcriptase-polymerase chain reaction (qRT-PCR) [79]. Sperm hsa-miR-34c-5p is the only noncoding RNA in semen that has a significant predictive value for male idiopathic infertility receiving ICSI cycles, according to one study. In addition, 10 miRNAs in total, including a panel of three, have shown good to exceptional diagnostic potential, particularly for non-obstructive azoospermia and asthenozoospermia [62]. Several studies documented SP and sperm piRNAs’ diagnostic potential, with findings suggesting a predictive utility for separating sperm quality states [80]. 

After ICSI, the piRNAs expressed in sperm are associated with the sperm concentration and fertilization rates, but no association was found with male fertility treatment or restoration of male sperm quality. PiR-58527 represents a helpful biomarker for distinguishing between nonobstructive and obstructive conditions. Through RNA modification and interference, small noncoding RNAs, such as transfer RNAs (tRNAs), regulate gene expression. Although SP encloses fragments produced from tRNA, their purpose remains unknown [60]. 

In SP, there is a high concentration of glycosylated proteins that are necessary for spermatogenesis, sperm maturation, capacitation, and fertilization [81]. Six glycoproteins have been found to be potential biomarkers for ART outcome prediction, including presepsin, CA-19.9 and CA-195 levels, and glycodelin-A [82]. According to research, glicoldelin keeps spermatozoa immobile and lowers cholesterol influx [83]. Significantly higher levels of SP CA-125 were identified in men with severe oligoasthenozoospermia; however, no correlations with clinical pregnancy rates or fertilization were found [84].

Nevertheless, a statistically significant connection was found when men were categorized based on their rate of fertilization (<66% or ≥66%) [85]. Soluble CD14 (sCD14-ST) levels increased during inflammation, but no correlation was found between them and the outcome of IVF or ICSI. Higher quantities of SP protein were found in men with high rates of fertilization and fertility [10]. 

Soluble CD147 is present in mouse spermatozoa and may play a role in sperm motility and the acrosome response. It has been suggested that it has a role in the fertilization of CD147 expressed in mouse cumulus cells. Chen et al. (2021) assessed the soluble CD147 levels in SP, as well as the IVF fertilization rate and pregnancy outcome. They found a positive correlation between fertility rate and serum plasma soluble CD147. Nevertheless, the SP of males in couples that did not become pregnant after IVF showed lower levels of CD147 [81].

A total of 32 semen protein indicators linked to various aspects of sperm quality, functioning, and fertilization potential were reported in 17 different investigations. Two important biomarkers were testis-expressed protein 101 (TEX101) SP levels and the enzymatic activity of N-acetyl-β-D-hexosaminidase [82,83].

Ejaculate fertility may be predicted by a number of proteins found in sperm and SP, including BAG6, HIST1H2BA, PON-1 activity, and CYP24A1. Using semen analysis, these proteins’ AUCs, which range from 0.921 to 0.950, assist clinicians in differentiating between fertile, subfertile, and infertile patients [61]. To determine their diagnostic usefulness in the diagnosis of subfertility, more investigation is required. Other semen proteins have also been found, including uPA, presepsin, and CATSPER1, albeit their prognostic potential for ART effectiveness is limited [60]. With respect to idiopathic male infertility, TEX101 have not shown any meaningful prognostic value. Despite inconclusive findings, these proteins remain potential biomarker candidates for predicting male (in)fertility [84].

Male fertility problems include anomalies in the testicles, vasectomy, and non-obstructive or obstructive azoospermia can be predicted with the help of semen protein indicators [24]. Serum plasma levels of α-glucosidase, CRISP1/PAP, and TEX101 enable clinicians to differentiate between these clinical conditions [85]. Seminal plasma levels have respective AUCs of 0.670, 0.929, and 0.609. Furthermore, the results of surgical procedures like vasectomy and testicular sperm extraction can be predicted by these indicators. To improve diagnosis and prognosis, more clinical trial testing of these biomarkers is required. 

Proteins in SP are involved in immune response regulation, spermatozoa protection, sperm maturation, capacitation, and the acrosome reaction. Prostate-specific antigen (PSA), clusterin, and epididymal secretory protein E1 (NPC2) were among the nine potential SP biomarkers that were investigated in seven investigations. PSA, which is produced only by the prostate and breaks down semenogelin and fibronectin, is necessary for the liquefaction of semen. According to one study, the quantity of galectin-3 bound to SP-derived EV and the rate of IVF fertilization were significantly positively correlated [86].

Normal male fertility depends on urokinase-type plasminogen activator (UPA) [87]. Animals with UPA downregulation present reduced sperm motility and fertility [88]. Total and active UPA levels were considerably higher in the pregnant group compared to the non-pregnant group in a study that investigated the association between these parameters and the clinical pregnancy outcome after ICSI. However, no discernible variations were found in the levels of α-glucosidase or SP creatine kinase [89].

Metabolomics represents a possible biomarker of sperm quality and fertilization potential [90]. A highly successful biomarker for male infertility problems such as idiopathic infertility, oligozoospermia, and sperm physiologic status is seminal plasma metabolomic fingerprint analysis. Lipid hormones, fatty acids, and ceramides showed good prognostic value for sperm recovery in patients with non-obstructive azoospermia. With remarkable sensitivity and specificity, the total nitroblue tetrazolium in the ejaculate, normalized by the sperm concentration, indicates the quality of the sperm. Solid diagnostic importance of ROS, TAC, ORP, metabolite profiles, and individual lipids in sperm and SP is attested to by research on male sperm quality and fertility concerns. The utilization of this technology in reproductive clinics may be hindered by the requirement for costly apparatus and skilled personnel [91]. 

Semen quality and male reproductive problems can be diagnosed and treated with the help of molecular biomarkers found in semen. Omics techniques, such as transcriptomics, metabolomics, proteomics, genomes, and DNA integrity and structure, may be crucial biomarkers [90]. Fertility clinics can benefit from this dependable, noninvasive, time- and money-saving method of diagnosing male reproductive problems. The detection of infertility etiologies may be accurate and cost-effective if molecular biomarkers are found using omics techniques [60]. To accurately forecast the physiologic condition of sperm and/or fertility, data integration with each omics area is essential. While systems biology approaches are still being investigated, the use of independent biomarkers is still in its infancy. Delivering real benefits to IVF institutions is the goal. There are benefits and drawbacks to omics research [92]. ROC analysis is an objective statistical method used to assess how accurate biomarkers are as diagnostic tools. Some studies, though, had tiny sample numbers and lacked important details. The absence of research employing ROC and OR/RR to demonstrate the relationship between biomarkers and clinical outcomes highlights the necessity for additional clinical trials [93].

Research employing metabolomics involves identifying and quantifying every low molecular weight substance found in biological systems, such as hormones and secondary metabolites [94]. Analyzing the seminal plasma metabolome helps in better understanding male infertility. The components of the sperm metabolome are lipids, carbohydrates, organic acids, amino acids, and biogenic amines [95]. For energy and to maintain pH and coagulation, spermatozoa need citric acid and fructose. Spermatogenesis requires glutamine and arginine, and lipid content and acyl-carnitines affect sperm motility [1]. Thus, metabolites are correlated with sperm motility, energy consumption, and metabolic activity. Moreover, metabolites are necessary for a number of procedures related to fertilization, implantation, and embryonic development. Reproductive biology research employs several techniques in metabolome analysis, including Fourier transform infrared spectroscopy (FTIR), mass spectrometry (MS), near-infrared (NIR), Raman, and proton nuclear magnetic resonance (1H NMR) [96]. Metabolomics techniques have been applied as noninvasive procedures to enhance the evaluation of embryo quality. Furthermore, metabolomics may be able to identify biomarkers linked to both male and female infertility. Certain metabolites linked to male gamete activity may be present or may fluctuate over time, thereby enabling evidence-based strategies to prevent or reduce infertility [97].

A metabolic technique using Raman spectroscopy has facilitated the diagnosis of normozoospermic and asthenozoospermic males. Furthermore, in the serum and SP of bulls with high and poor fertility, 1H NMR has found biomarkers associated with fertility. Whereas SP contains metabolites such as citrate, tryptamine, taurine, and leucine, serum contained isoleucine, citrulline, asparagine glycogen, and taurine [92]. Hamamah et al. used 1H NMR to determine that males with spermatogenic failure had greater ratios of choline to citrate, choline to lactate, and glycerophosphorylcholine to choline in their SP than those with obstructive azoospermia. Males with normozoospermic infertility have urine that contains a number of small molecular markers, which have been identified using liquid chromatography–mass spectrometry (LC–MS), bioinformatics, and multivariate analyses [98].

## 12. Limitations and Challenges

Although SP offers potential diagnostic significance, it is rarely evaluated in the clinic. The intricate nature of male infertility, standardization problems, and confounding variables make the results hard to interpret. Determining clinically meaningful cut-off levels and resolving conflicting views regarding the interpretation of SP indicators require additional research. The lack of sophisticated clinically validated instruments makes seminal plasma analysis an uncommon diagnostic method for male infertility. Recognizing and removing these limitations and obstacles is essential to maximizing its therapeutic efficacy. By overcoming these barriers, researchers can create more efficient techniques for both diagnosis and therapy.

## 13. Conclusions and Future Directions

Male reproductive system problems have few accurate diagnostic techniques. Male infertility-related genetic markers can be found via next-generation sequencing. High-throughput proteomics and whole-exome sequencing can identify uncommon genetic variations that affect the quality and functionality of sperm. New biomarkers can be found by measuring alterations in the seminal plasma of male infertility patients. It is possible to look at genomic, epigenomic, transcriptomic, and proteomic variations in order to identify specific genomic modifications associated with infertility. Alterations in signal transduction pathways, noncoding RNA regulation, and epigenetic control may have a direct or indirect impact on these modifications. By focusing on particular mRNAs, testis-specific miRNAs control spermatogenesis and male fertility. It is essential to provide a noninvasive technique for determining the prognosis, grading the varicocele, and identifying male infertility. Researchers could monitor changes in seminal plasma biomarkers over time and in response to different therapies by carrying out extensive, longitudinal cohort studies. Translating research findings into clinical practice requires the development of standardized protocols for biomarker assessment, including sample collection, processing, and analysis (Table 2).

## Figures and Tables

**Table 1:** Factors influencing the seminal plasma and their mechanism of action.

Factors	Mechanism of Act
Genetics	Genetic variables such as predisposition to specific medical disorders that may have an impact on semen quality.
Age	Age-related changes in hormone levels and general health can impact the composition of SP.
Lifestyle factors	Stress, drug use, alcoholism, smoking can have a negative impact on the consistency of SP.
Medications	Certain drugs may alter hormone levels or metabolic processes, which may have a direct or indirect effect on SP.
Diet and nutrition	SP composition can be positively impacted by a well-balanced diet high in minerals including zinc, selenium, vitamin C.
Health conditions	SP consistency can be impacted by a number of medical problems, including infections, inflammation, hormone abnormalities, and metabolic disorders.
Environmental Toxins	Exposure to heavy metals, herbicides, environmental contaminants, and other factors can affect SP.
Sexual activity	Intensity and duration of sexual stimulation might affect SP composition.
Frequency of ejaculation	Longer intervals without sperm are typically linked to higher concentrations of sperm.
Hydration status	Dehydration can impact SP volume and consistency.
Exercise	Exercise intensity and duration are two important variables that can affect the impact of regular physical activity on SP composition.

**Table 2:** The table provides seminal plasma indicators along with the mechanism of action.

Seminal Plasma Biomarkers	Mechanism of Action
Prolactin-induced protein (PIP)	Ability to inhibit the immunological system and its presence in seminal fluid. May aid in liquefying fibronectin and lessening the effects of ASA, which would aid in the fertilization process.
Seminogelin I (SgI) isoform b preprotein Seminogelin II (SgII) precursor	The coagulum formed by freshly ejaculated human semen contains prominent proteins. These proteins, with the exception of the epididymis, which produces a little quantity of SgII, are primarily found in seminal vesicles and are missing from other tissues.
Albumin preproprotein	It modifies the lipid composition of the sperm plasma membrane, possibly by lipid exchange or hydrolysis. The presence of albumin in the adult human testis and SP may be linked to its potential to transport androgens.
Prostate specific antigen isoform 1 preproprotein (PSA)	PSA is a type of serine protease that dissolves semen to facilitate spermatozoa development. In healthy males, relatively low levels of PSA (<1 ng/mL) are detected in the blood.
Epididymal secretory protein E1 precursor	May have an impact on sperm motility; in some species, phosphoglycerate kinase is also connected to sperm motility. Binds to the surface of sperm, especially in the acrosome and midpiece.
Lactotransferrrin precursor	Forms a significant portion of the antigens coating sperm by binding to them.
Prosaposin isoform a preproprotein	Pre-fertilization stages of the fertilization process involve the immunoglobulin super-family (IgSF) member IZUMO4.
Tissue inhibitor of metalloproteinase (TIM)1 precursor	Between remodeling and ECM breakdown, TIM1 and TIM2 maintain the balance. Multiple bioassays show that different effects of multifocal proteins prevent angiogenesis.
Armadillo—repeat protein (Armc8)	Part of the LisH / CTLH complex, also known as the glucose-induced degradation deficient (GID) complex, is responsible for controlling the FBPase degradation process. This multi-subunit E3 ligase complex → involvement in basic pathways regulating development and homeostasis in multiple species.
Fibronectin (FN) 1 isoform 3 preproprotein	Present on the surface of sperm, multifunctional diametric glycoprotein is essential to sperm-oocyte contact and fertilization.
Cathepsin D (CTSD) preprotein/preproprotein	Aspartic protease is expressed by lysosomes and endosomes. An essential enzyme that changes the sperm’s plasma membrane and enhances fertilization.
Zinc—alpha—2—glycoprotein 1	To control insulin sensitivity and to enhance glucose utilization and lipid metabolism.
Acid phosphatase (AP), prostate short isoform precursor	Both the development of the ability to fertilize and the quiescence of spermatozoa till ejaculation are impacted by AP.
Cystatin S precursor Cystatin C precursor	The spermogram’s evidence is modified by motility and numbers. Clinical suspicions of infertility and subfertility may exist at first.
Ubiquitin and ribosomal protein S27a precursor	Some proteins that are unique to male germ cells and required for the generation of sperm are folded under cotranslational regulation.
Carboxypeptidase E preproprotein (CPE)	Pro-hormone sorting receptor and processing enzyme that operates inside cells. Spermatozoa with high motility had lower CPE abundances. Sperm motility declined due to CPE inhibition in spermatozoa, and throughout the capacitation process, acrosome exo-cytosis and tyrosine phosphorylation declined as expected. These changes resulted in a considerable drop in the cleavage rate during IVF, which was accompanied by a decrease in intracellular Ca^2+^.
Clusterin (CLU) isoform 1	In the female reproductive system, essential SP glycoproteins are important for both sperm capacitation and immunological tolerance.
Extracellular matrix protein 1 (ECM1) isoform 1 precursor	Spermatozoa are able to secrete genes that are expressed by the epididymis. In spermatozoa and Sertoli cells, it specifically aids in controlling the spermatogenesis process.
Beta 2 microglobulin (B2M) precursor	Split ejaculates, which were selected based on the electrophoretic pattern found for each component, indicate that the B2M in SP is sourced equally from the prostate and seminal vesicles. A portion of bound B2M is present in human sperm in addition to free B2M in SP.
Galectin 3 binding protein	Derived EVs and fertilization rates have a positive correlation. The transfer of galectin 3 to the sperm surface during post-testicular maturation in EVs is crucial for the binding of spermatozoa to the zona pellucida following capacitation.
Orosomucoid 1 (ORM1) precursor Orosomucoid 2 (ORM2)	Several key regulatory mediators collaborate to alter the expression of ORM genes, including glucocorticoids, interleukin (IL)-1, TNF-α, and IL-6. Acute-phase reactant and disease marker roles, immunological modulation, drug binding and transport, capillary barrier maintenance, and sphingolipid metabolism mediation are just a few of the roles it plays.
Prostaglandin (H2) D—isomerase—1 peptide	Sperm transit within the female reproductive canal is facilitated by the induction of sperm motility and peristaltic concentrations (PGF2a).
DJ—1 protein	Multifunctional protein essential to tissues with higher order biological processes, such as the testis and brain. Male fertility is associated with DJ-1, which causes sperm counts to decrease in response to exposure to sperm toxicants.
Secretory leukocyte peptidase inhibitor precursor (SLP1)	Accountable for the external serum’s anti-HIV characteristics.
Cathepsin B (CTSB) preproprotein	One of the cysteine protease family members. Essential elements of sperm development and spermatogenesis in humans.
Serine proteinase inhibitor, clade A, member 1	Enzymes that are hydrolytic in nature and are involved in spermatogenesis, sperm capacitation, travel within the female reproductive canal, recognition and binding with the egg’s zona pellucida, the acrosome reaction, and the fusing of sperm and egg.
Transferrin	Reliable marker of tubular seminiferous function. An unselected group of infertile individuals with reduced sperm concentration and/or motility showed a correlation between seminal transferrin and sperm count.
Macrophage migration inhibitory factor—1 peptide	Originate in the testicular interstitium, a vital auxiliary cell in the process of spermatogenesis. The testicular stroma cells interact with such cells and other supporting cells to maintain the microenvironmental stability of those cells.
Protein tyrosine phosphatase, receptor type, sigma isoform 1 precursor	Associated with acquiring the hyperactive motility needed by spermatozoa to pierce the oocyte’s cumulus and zona pellucida.

## Data Availability

Data are unavailable due to privacy or ethical restrictions.
